# Assessing Barriers to Effective Coverage of Health Services for Adolescents in Low- and Middle-Income Countries: A Scoping Review

**DOI:** 10.1016/j.jadohealth.2020.12.135

**Published:** 2021-10

**Authors:** Elizabeth K. Stierman, Anna Kalbarczyk, Htet Nay Lin Oo, Theadora Swift Koller, David H. Peters

**Affiliations:** a Department of International Health, Bloomberg School of Public Health, Johns Hopkins University, Baltimore, Maryland; b Bloomberg School of Public Health, Johns Hopkins University, Baltimore, Maryland; c Gender, Equity and Human Rights Team, World Health Organization, Geneva, Switzerland

**Keywords:** Health services accessibility, Services utilization, Effective coverage, Health equity, Adolescent health services, Low- and middle-income countries

## Abstract

**Purpose:**

Understanding barriers to health services, as experienced by adolescents, is important to expand effective and equitable coverage; however, there is limited discussion on methods for conducting barrier assessments and translating findings into action.

**Methods:**

We conducted a scoping review of literature published between 2005 and 2019 on barriers to health services for adolescents in low- and middle-income countries. The review was guided by a framework that conceptualized barriers across multiple dimensions of access (availability, geographic accessibility, affordability, and acceptability), utilization, and effective coverage.

**Results:**

We identified 339 studies that assessed barriers related to at least one dimension of the operational framework. Acceptability (93%) and availability (88%) of health services were the most frequently studied access dimensions; affordability (45%) and geographic access (32%) were studied less frequently. Less than half (40%) of the studies evaluated utilization, and none of the 339 studies assessed effective coverage. Attention to equity stratifiers (e.g., income, disability) was limited. Topics studied reflected only a subset of the major causes of adolescent death and disability.

**Conclusions:**

Holistic, equity-oriented approaches are needed to better understand barriers across multiple dimensions that together determine whether health services are accessible, used, and effectively meet the needs of different adolescent groups.

**Implications and Contribution**

This review highlights the need to examine barriers systematically and from the perspective of adolescents rather than for selected services that neglect major health burdens. The findings demonstrate the need to study equity and the intersection of multiple forms of disadvantage and for researchers to work more closely with decision-makers.

Worldwide, there are an estimated 1.2 billion young people between the ages of 10 and 19 years [1]. Adolescents account for one sixth of the world population, and in many low- and middle-income countries (LMICs), they comprise an even higher proportion of the country's population. Adolescence is a critical phase for physical, social, cognitive, and emotional development, and the health needs and risks faced by adolescents are distinct from younger children and adults [2]. The most common causes of death among adolescents include road traffic injuries, infectious diseases, interpersonal violence, self-harm, and, among adolescent girls, complications related to pregnancy and childbirth [1].

Increasingly, adolescence has gained attention as a critical period because of its importance in laying the foundation for future health as children transition to adulthood. This recognition has led to the launch of the *Global Strategy for Women's, Children's and Adolescents' Health* in 2015 [3], creation of a *Lancet Commission on Adolescent Health and Wellbeing* [2], and development of guidance by the World Health Organization (WHO) for *Global Accelerated Action for the Health of Adolescents*, widely known as AA-HA! [4]. Adolescence is also an important window of opportunity for tackling the transgenerational transmission of health inequities, as trajectories for health across the life course (including exposure to risk factors and adverse social determinants of health) can be further defined during adolescence.

Adolescents often face greater legal and financial barriers to accessing health services than other age groups, and access may be further constrained by social norms, requirements for parental consent, or health providers' beliefs and attitudes on the appropriateness of providing certain services to adolescents of a certain age, sex, gender, or marital status [5]. At the global level, despite substantial progress in reducing the overall number of adolescent deaths, inequities in adolescent health outcomes persist. The mortality rate among adolescents living in the bottom quintile of countries ranked by Socio-Demographic Index was 95 deaths per 100,000 in 2017, over four times the rate in the top quintile (22 deaths per 100,000) [6,7].

Health and socioeconomic crises, such as the current crisis brought on by COVID-19, can exacerbate risks to the well-being, safety, and health of adolescents, as well as the barriers they face in accessing health services. An increase in sexual exploitation, sexual abuse, teenage pregnancy, and early marriage was reported during the Ebola outbreak in West Africa [[8], [9], [10]]. Crises can also adversely impact adolescent's mental health [11,12]. Furthermore, school closures—while a strategy for minimizing the spread of communicable diseases—can reduce access to school meals and school-based health services [8,13,14].

To support efforts to ensure that no adolescent is left behind in progress toward the Sustainable Development Goals, WHO has developed the *Handbook for conducting an Adolescent Health Services Barriers Assessment (AHSBA) with a focus on disadvantaged adolescents* [5]. This scoping review was part of a WHO program to provide guidance for national and subnational health authorities on conducting barrier assessments and incorporating a focus on health inequities and their drivers within existing monitoring and evaluation systems.

The purpose of this scoping review is to identify existing methods to assess barriers on the pathway from access to effective coverage of health services for adolescents in LMICs. The specific objectives were to (1) identify which dimensions of disadvantage, types of barriers, and health services/conditions are most frequently studied in the literature; (2) review existing quantitative, qualitative, and mixed methods to assess barriers to effective health coverage among adolescent populations; and (3) examine how results of studies on adolescent health barriers are used.

We applied an “operational framework for barriers on the pathway from access to effective coverage” (Table S1 in the Appendix) to guide the review, drawing on the Tanahashi framework on effective coverage [15] and other key frameworks and concepts relevant to equity-oriented, rights-based, and gender-transformative health system strengthening and intersectoral action for health [[16], [17], [18], [19], [20]] (Table S1 in the Appendix). Access is influenced by supply- and demand-side factors across four dimensions: availability, geographic accessibility, affordability, and acceptability. Utilization is a measure of actual use and is a product of these dimensions of access. Effective coverage is the proportion of the population who need a health service that receive care satisfactory to achieve the desired health effect [15,21]. For coverage to be effective, it first requires access and utilization and, in addition, depends on service quality and patient adherence. Effective coverage is also an estimate of the fraction of potential health gain that is actually delivered to the population through the health system. Note the term “effective coverage” is used throughout the article in line with this definition; it should not be confused with insurance coverage as the term is commonly used in the U.S.

## Methods

The protocol followed the Preferred Reporting Items for Systematic Reviews and Meta-analysis extension for Scoping Reviews (PRISMA-ScR) guidelines [22] and was registered with the Open Science Framework (https://osf.io/v9ke3).

To identify published literature on barriers to care for adolescent populations in LMICs, two databases were searched from 2005 to 2019: PubMed and Embase. These two databases were selected because of their comprehensive representation of the peer-reviewed, published literature on this subject. Search strategies were drafted by a global health informationist and refined through team discussion. Five core search concepts were selected with their Medical Subject Headings terms and vocabulary equivalents in addition to related key search terms: Adolescent/Child, Developing Countries, Access Barriers, Health Services, and Evidence. For example, the search concept of Access Barriers included Medical Subject Headings terms for “Health Services Accessibility” and “Health Equity” in addition to a range of search terms related to access, equity, affordability, stigma, cultural norms, and barriers. Exclusion filters were used to remove literature on children under five and maternal health (without reference to adolescents). The initial search was performed on September 26, 2018, with an update on October 14, 2019, and there was no restriction on the language of publication. The final search strategy is provided in Supplementary File 1.

**Figure 1:**
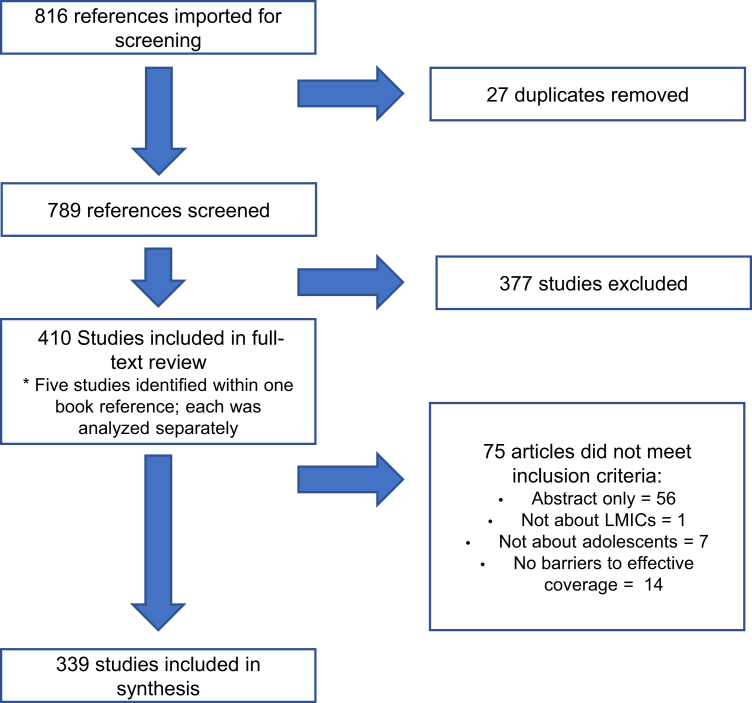
PRISMA diagram for scoping review.

A total of 816 eligible references were imported into an electronic database for title and abstract screening and full-text review (Figure 1). Twenty-seven duplicate articles were removed; each remaining imported reference (n = 789) underwent a title and abstract screening. Two reviewers screened each reference based on the following exclusion criteria: not an LMIC (n = 75), not about adolescents (n = 86), did not mention a barrier to access (n = 202), not a full-text article (n = 3), ongoing study (n = 71), a protocol only (n = 32), and an opinion piece only (n = 63). Some articles met more than one criterion. Discrepancies were resolved by a third, final reviewer, yielding 377 references that were excluded.

In total, 410 articles remained eligible for full-text extraction. One reference included five unique stand-alone studies in separate chapters, and each of these were analyzed separately. The final number of articles that underwent full-text extraction was 414. The data from each article were then extracted by two independent reviewers using a standardized form based on the operational framework. A third consensus reviewer compared data entries and made final determination in case of any discrepancies. During this stage, 75 articles were excluded for not meeting the inclusion criteria: abstract only (n = 56), not about LMICs (n = 1), not about adolescents (n = 7), and no barriers to effective coverage (n = 14); some articles met more than one exclusion criteria.

Data extracted during the full-text review was charted along the pathway from access to effective coverage based on our operational framework; variables included supply- and demand-side barriers related to the four access dimensions (availability, geographic accessibility, affordability, and acceptability), as well as utilization and effective coverage. Other key variables included dimensions of disadvantage, inequality metrics, type of researchers, target audiences, and how the results were disseminated and used. Data on study characteristics included region of study, study purpose, health service/condition(s) addressed, whether an intervention was assessed, setting, scale, data source(s), duration of data collection, and study method(s).

The data extracted from the studies were reviewed for discrepancies across response categories and recoded as appropriate. For example, some data coded as “other” was recategorized into existing categories. Univariate and bivariate analyses of data were then performed.

## Results

### Description of studies

The review identified 339 unique studies meeting the inclusion criteria (Supplementary File 2). Study characteristics are shown in Table 1. Geographically, Africa was the most studied region (75%), followed by Southeast Asia (17%), Western Pacific (10%), the Americas (9%), Eastern Mediterranean (5%), and the European Region (4%); among these, 11% studied multiple countries. More than half of the studies addressed sexual and reproductive health services (56%); HIV/AIDS (31%) and mental health (17%) were also common topics. Very few studied primary care or more broadly defined health services, and 85 (25%) studies assessed an intervention to improve a dimension of access. Studies were implemented across all scales: national (30%), state/province (13%), district/county (23%), municipality/city (27%), and village/community (7%).

**Table 1:** Characteristics of articles (n = 339)

	Number (%)^a^
Region of study	
Africa Region	253 (75%)
Southeast Asia Region	57 (17%)
Western Pacific Region	34 (10%)
Region of the Americas	30 (9%)
Eastern Mediterranean Region	17 (5%)
European Region	15 (4%)
Multiple countries/regions	38 (11%)
Health service/condition	
Sexual and reproductive health	190 (56%)
HIV/AIDS	106 (31%)
Mental health	57 (17%)
Sexual violence	24 (7%)
Cervical cancer/HPV vaccine	19 (6%)
School health	19 (6%)
Health promotion/behavior change	15 (4%)
Outpatient, general (e.g., primary care provision)	14 (4%)
Noncommunicable and chronic diseases	9 (3%)
Health screening	6 (2%)
Environmental health	5 (1%)
Other	26 (8%)
Intervention assessed	
Intervention (policy, program, project)	85 (25%)
No intervention	254 (75%)
Scale of study	
National	100 (30%)
Municipality/city	91 (27%)
District/county/parish/ward	78 (23%)
State/province	45 (13%)
Village/community	25 (7%)
Dimensions of disadvantage/vulnerability/inequity stratifiers assessed	
Sex	234 (69%)
Rural/urban	104 (31%)
Socioeconomic status	99 (29%)
Marital status	63 (19%)
Income	62 (18%)
Education	54 (16%)
Geography (other than rural/urban)	51 (15%)
Age	46 (14%)
Out-of-school youth	37 (11%)
Orphans/living arrangements	34 (10%)
Occupation/employment status	33 (10%)
Religion	22 (6%)
Parity/childbearing status	20 (6%)
Ethnicity/race	16 (5%)
Youth in displaced populations	13 (4%)
Developmental disability	12 (4%)
Physical disability	9 (3%)
Youth living in conflict areas	10 (3%)
Homeless/informal housing	10 (3%)
Youth in sex industry	7 (2%)
Substance abuse	7 (2%)
Sexual orientation	7 (2%)
Youth living in remote areas	5 (1%)
Gender identity	5 (1%)
Other	15 (4%)

a Multiple responses are possible per category.

### Barriers to access and use

The literature presents a diverse range of disadvantages and equity stratifiers that could influence adolescents' access to health services, but few dimensions except for sex (i.e., differences between adolescent boys and girls) were consistently assessed across studies. As shown in Table 1, sex was considered in 69% of studies. However, although many studies reported sex-disaggregated data, few studies considered the influence of gender norms, roles, or relations on access. Only five (1%) studies explored how gender identity, and only seven (2%) studies explored how sexual orientation may impact adolescents' access to health services. After sex, the dimensions most frequently studied include rural/urban residence (31%), socioeconomic status (29%), marital status (19%), income (18%), education (16%), and geography (15%). Only 11% of studies considered disadvantages faced by out-of-school youth.

**Figure 2:**
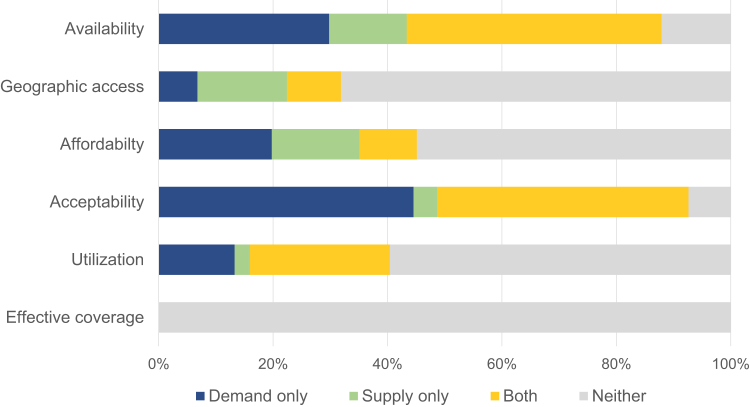
Dimensions of access, utilization, and effective coverage identified by studies in relation to supply and/or demand for adolescent health services.

Supply-side barriers arise from constraints in the provision of services, whereas demand-side barriers arise from constraints at the individual, household, or community level [23]. Most studies (69%) identified both supply-side and demand-side barriers to effective coverage of health services for adolescents. Of those remaining, 88 (26%) assessed only demand-side barriers, and 18 (5%) focused on only supply-side barriers. Acceptability (93%) and availability (88%) of health services were the most frequently studied dimensions of access, followed by affordability (45%) and geographic access (32%; Figure 2). Less than half (40%) of the studies evaluated the utilization of services. No article assessed effective coverage directly.

We also examined the intersection between dimensions of access, utilization, and effective coverage (e.g., availability) and equity stratifiers (e.g., place of residence; Table S2 in the Appendix). Patterns were generally similar across dimensions. However, rural/urban residence and geographic considerations were more often considered in studies addressing geographic accessibility or utilization. Socioeconomic status and income were more often analyzed in studies of affordability, utilization, and geographic accessibility. In Table 2, we show how studies of different health conditions varied in their consideration of access, utilization, and effective coverage dimensions, highlighting how, in each case, most of the studies focused on dimensions of availability and acceptability.

**Table 2:** Frequencies that health coverage dimensions were studied by health condition/service

	Availability	Accessibility (geographic)	Affordability	Acceptability	Utilization	Effective coverage
Sexual and reproductive health, n = 190	92%	33%	45%	92%	49%	0%
HIV/AIDS, n = 106	82%	25%	29%	96%	35%	0%
Mental health, n = 57	68%	19%	42%	98%	26%	0%
Sexual violence, n = 24	83%	33%	50%	100%	46%	0%
Cervical cancer/HPV vaccine, n = 19	89%	63%	63%	84%	37%	0%
School health, n = 19	100%	42%	47%	89%	58%	0%
Health promotion/behavior change, n = 15	100%	47%	53%	73%	53%	0%
Outpatient, general (e.g., primary care), n = 14	100%	36%	71%	93%	50%	0%
Noncommunicable and chronic diseases, n = 9	89%	33%	78%	89%	0%	0%
Health screening, n = 6	100%	33%	50%	83%	67%	0%
Environmental health, n = 5	100%	80%	80%	80%	20%	0%

Percentages are row percentages.

### Methods for assessing barriers

Qualitative methods were used in 236 (70%) studies, and quantitative methods were used in 186 (55%) studies (Table 3). Among these, 83 (24%) used mixed or multiple methods that included at least one quantitative and one qualitative method. Interviews and focus group discussions were the most common qualitative methods used. Surveys were the most common quantitative method. Among quantitative studies, 88 (47%) presented inequality metrics, such as ratios or regression coefficients, to compare or adjust for differences between groups. Most studies were conducted by researchers from academic organizations (95%), either solely or sometimes in collaboration with researchers from government or civil society. Only 199 (59%) studies reported the duration of data collection; among these, the median duration was 4 months (interquartile range: 2–9 months).

**Table 3:** Methodology used in articles (n = 339)

	Number (%)^a^
Methods used	
Any qualitative	236 (70%)
Any quantitative	186 (55%)
Mixed methods/multimethod^b^	83 (24%)
Qualitative method used	
In-depth interviews	145 (43%)
Focus group discussions	121 (36%)
Document review	46 (14%)
Observation	11 (3%)
Workshop or meeting	7 (2%)
Narratives	7 (2%)
Case study	5 (2%)
Other	20 (6%)
Quantitative method used	
Survey of participants, students, or another target group	106 (31%)
Household survey	38 (11%)
Systematic reviews	19 (6%)
Health facility survey	17 (5%)
Review of quantitative data/records	14 (4%)
Other	18 (5%)
Inequality metrics used (n = 186)^c^	
Range/ratio (relative odds, relative risk)	66 (35%)
Regression coefficients	24 (13%)
Other	12 (6%)

a Multiple responses are possible; sum of category subtotals may exceed total number of articles. Percentages are calculated out of total number of articles, n = 339.

b Mixed methods/multimethod include studies with at least one quantitative and one qualitative method.

c Percentages for inequality metrics are calculated out of total number of quantitative studies, n = 184.

### Dissemination and use of results

Few studies (18%) identified a target audience for their research, and it was often unclear if and to whom results were disseminated. Beyond the academic or research community, the most common targets were, in order, governments, civil society organizations, implementing agencies, and funding organizations. A total of 104 studies (31%) explicitly described how results would be used. The most common uses were to inform the design or planning of a program (19%), process improvement or problem-solving for service delivery (15%), advocacy related to the population under study (8%) and to influence policy (6%) and policy agenda setting (6%).

## Discussion

Our scoping review sought to identify barriers along the pathway from access to effective health coverage among adolescent populations in LMICs. Notably, none of the 339 studies identified in our search of the adolescent health literature assessed effective coverage directly. In 1978, Tanahashi called for measures of coverage that go beyond “contact coverage” to consider whether services are adequately meeting people's needs [15]. More recently, WHO, Organization for Economic Cooperation and Development, the World Bank, and the Lancet's Commission on High-Quality Health Systems in the SDG Era have echoed the call for better quality of care to reduce mortality and improve population health, with effective coverage as a necessary measure of a health system's performance [24,25]. Yet, our review suggests that research on barriers to health services faced by adolescents focuses primarily on access to services without considering measures of technical quality of care or treatment adherence that is needed to determine effective coverage.

The selection of topics and their narrowness suggest that more could be done to better prioritize a research agenda on barriers to adolescent health. Most studies focused on specific diseases, such as HIV/AIDS, or specific services, such as reproductive and sexual health services. Only a single study addressed injuries, although injuries are the leading cause of death among adolescents [26]. Other important causes or risk factors for adolescent morbidity and mortality, including diarrhea, respiratory infections, nutrition, and substance use, were absent in the literature.

Overall, little attention was given to the diverse and multiple health needs of adolescents in this critical phase of development and as they transition to adulthood. Building on the movements for primary health care and universal health coverage, the WHO and the United Nations Children's Fund have emphasized the importance of people-centered and integrated health services that respond to the holistic needs of individuals across their life course [27,28]. Our scoping review indicates that the dominant paradigm in adolescent health service research is still one focused on a single disease or condition.

Certain groups of adolescents may face greater barriers to accessing services, but few studies considered dimensions of disadvantage (or equity stratifiers) beyond sex, geography, or socioeconomic factors. Schools are a relatively common setting for studies (21%) and a common platform for delivering health services to adolescents in many countries. Yet secondary school attendance is far from universal in many low-income countries, and little attention has been given to studying barriers for out-of-school youth (11% of studies). There remain significant disparities across adolescents in different wealth quintiles [29]. Fewer than 5% evaluated how disability, gender identity, or sexual orientation might impact access. Only a few studies considered the impact of displacement, migration, or conflict. The reasons for the underrepresentation of these populations in the literature may arise from difficulties in accessing and studying these populations. For example, in some countries, research efforts to understand barriers faced by lesbian, gay, bisexual, transgender, or intersex adolescents may be constrained by legal barriers, such as antihomosexuality laws [30]. In addition, disaggregated data collected through national surveys and other quantitative studies is often only available for a few stratifiers, such as biological sex, place of residence, education level, or wealth quintile.

Most of the studies focused on the availability and acceptability of health services, whereas geographic accessibility, affordability, and utilization were less frequently studied; this may partly reflect the relative feasibility of different methodological approaches. Studies on utilization, as well as those on effective coverage, usually require sampling from a representative population of adolescents who could benefit from a service, including both those who use and those who do not use the service at the present time. These studies more often use survey research methods. Conversely, among the 339 studies identified, qualitative methods were preferred; these tend to apply nonprobability sampling techniques to identify a smaller group of informants, frequently clients, or providers recruited from a health care setting or community program. This strategy can allow for rich discussion on topics related to service availability and acceptability, such as perceptions of service quality, staff motivation, and cultural norms. However, recruitment strategies that focus on current clients or program participants may miss the voices of those most underserved by the health system; it is possible that geographic access and affordability may be more salient concerns among those “missing” populations.

It is encouraging to see the broad range of research methods being used, including multiple research methods to generate distinct and complementary perspectives on the barriers faced by adolescents in accessing health services. The use of multiple methods can help construct a more comprehensive understanding of barriers on the pathway from access to effective coverage, even if the study is focused on a single condition or health service. However, 256 studies (76%) relied either solely on quantitative methods or solely on qualitative methods. Participatory techniques that engaged adolescents as collaborators in the research process were rarely used; only four studies used participatory action research techniques, which seek to engage those affected by the research in the generation, validation, and use of research evidence for action.

There are other areas for improvement as well. Although most of the researchers were from academic institutions, the articles rarely discussed how findings would be disseminated beyond the academic community. Funders can play an important role in requiring that researchers articulate a strong dissemination plan as part of the research proposal; they can also invest in funding modalities that encourage partnerships with civil society and implementing organizations. In addition, although many studies sought to answer questions related to national programs and policies, time and scale were often limited. The majority (57%) of studies were conducted at the district/county or lower level (e.g., city, village), and the duration of data collection was short, with a median of 4 months. Notwithstanding the applied nature of the type of research, it seems there are many missed opportunities to enhance the dissemination and application of results. More meaningful participation of adolescents and a greater collaboration with government, implementing agencies, and civil society organizations in the identification of topics and dissemination activities could enhance the impact of these studies.

### Limitations

The research was restricted to peer-reviewed literature. The findings may not reflect the full range of adolescent health issues explored in the gray literature. It also may not reflect the range of research conducted by civil society organizations, governmental bodies, and health service providers for policy, planning, and evaluation purposes that is not published in the peer-reviewed literature. The review was also limited by the lack of clear description of research methods in some articles. In addition, the results recorded as “other” in the data extraction form were analyzed, and a new category was created if there were at least five relevant responses. The value of these “new” categories may be underrepresented if reviewers were less likely to identify options that did not appear on the prespecified list. Geographically, three fourths of studies focused on Africa. As the region with the highest number of adolescent deaths, it is encouraging to see a high proportion of studies focused on the region. As a result, we acknowledge that findings may be less generalizable to other regions.

## Conclusion

This scoping review identified that there is much capacity to conduct assessments of barriers on the pathway from access to effective coverage of adolescent health services in LMICs. There is still a need to identify such barriers in a more holistic way, particularly from the perspective of adolescents, rather than a focus on single services or health conditions. Other neglected areas of study include injuries—globally the most frequent cause of adolescent death—and common infections in adolescence. Few studies considered barriers faced by out-of-school youth, adolescents with disabilities, and other groups that may face additional challenges in accessing health services. The lack of effective coverage assessments in adolescent health is not unique to this part of the lifespan but represents another area where further research is needed. More effort is needed on deepening the understanding of equity in examining barriers to adolescent health services, looking beyond single indicators and toward the intersection of multiple forms of vulnerability and disadvantage. Finally, if research on barriers to adolescent health is to make a bigger impact on policy and practice, more effort is needed in engaging key groups beyond researchers in prioritizing topics and in the translation of research findings to practice. In the current COVID-19 health and socioeconomic crisis, there is a risk of particular threats to adolescent health and the potential for increased health inequities among this age group. Assessing barriers to health services experienced by adolescents is important to generate evidence for the prevention of widening inequities. Together these findings suggest that more critical reflection on the barriers to access is required moving forward.
