# Supplementary material for: Assessing Barriers to Effective Coverage of Health Services for Adolescents in Low- and Middle-Income Countries: A Scoping Review
Source: J Adolesc Health. 2021 Oct;69(4):541–8. doi: 10.1016/j.jadohealth.2020.12.135 (PMC8442758; doi:10.1016/j.jadohealth.2020.12.135)
Supplement: Supplementary File 1 [file mmc1.docx]

**Supplementary File 1: Search Strategy**

**PubMed: Initial Search 9/26/18; Update 10/14/2019 Dates Limited 2005-2019**

Concept #1: Adolescent/Child

“adolescent”[tiab] OR “adolescents”[tiab] OR “adolescence”[tiab] OR "youths"[tiab] OR "girls"[tiab] OR “youth”[tiab] OR “young people”[tiab] OR “teenage”[tiab] OR “teenager”[tiab] OR “teenagers”[tiab]

Concept #2: Developing Countries

"developing country"[tiab] OR "developing countries"[tiab] OR "developing nation"[tiab] OR "developing nations"[tiab] OR "developing population"[tiab] OR "developing populations"[tiab] OR "developing world"[tiab] OR "less developed country"[tiab] OR "less developed countries"[tiab] OR "less developed nation"[tiab] OR "less developed nations"[tiab] OR "less developed population"[tiab] OR "less developed populations"[tiab] OR "less developed world"[tiab] OR "lesser developed country"[tiab] OR "lesser developed countries"[tiab] OR "lesser developed nation"[tiab] OR "lesser developed nations"[tiab] OR "lesser developed population"[tiab] OR "lesser developed populations"[tiab] OR "lesser developed world"[tiab] OR "under developed country"[tiab] OR "under developed countries"[tiab] OR "under developed nation"[tiab] OR "under developed nations"[tiab] OR "under developed population"[tiab] OR "under developed populations"[tiab] OR "under developed world"[tiab] OR "underdeveloped country"[tiab] OR "underdeveloped countries"[tiab] OR "underdeveloped nation"[tiab] OR "underdeveloped nations"[tiab] OR "underdeveloped population"[tiab] OR "underdeveloped populations"[tiab] OR "underdeveloped world"[tiab] OR "middle income country"[tiab] OR "middle income countries"[tiab] OR "middle income nation"[tiab] OR "middle income nations"[tiab] OR "middle income population"[tiab] OR "middle income populations"[tiab] OR “middle-income settings”[tiab] OR “low-income settings”[tiab] OR "low income country"[tiab] OR "low income countries"[tiab] OR "low income nation"[tiab] OR "low income nations"[tiab] OR "low income population"[tiab] OR "low income populations"[tiab] OR "lower income country"[tiab] OR "lower income countries"[tiab] OR "lower income nation"[tiab] OR "lower income nations"[tiab] OR "lower income population"[tiab] OR "lower income populations"[tiab] OR "underserved country"[tiab] OR "underserved countries"[tiab] OR "underserved nation"[tiab] OR "underserved nations"[tiab] OR "underserved population"[tiab] OR "underserved populations"[tiab] OR "underserved world"[tiab] OR "under served country"[tiab] OR "under served countries"[tiab] OR "under served nation"[tiab] OR "under served nations"[tiab] OR "under served population"[tiab] OR "under served populations"[tiab] OR "under served world"[tiab] OR "deprived country"[tiab] OR "deprived countries"[tiab] OR "deprived nation"[tiab] OR "deprived nations"[tiab] OR "deprived population"[tiab] OR "deprived populations"[tiab] OR "deprived world"[tiab] OR "poor country"[tiab] OR "poor countries"[tiab] OR "poor nation"[tiab] OR "poor nations"[tiab] OR "poor population"[tiab] OR "poor populations"[tiab] OR "poor world"[tiab] OR "poorer country"[tiab] OR "poorer countries"[tiab] OR "poorer nation"[tiab] OR "poorer nations"[tiab] OR "poorer population"[tiab] OR "poorer populations"[tiab] OR "poorer world"[tiab] OR "developing economy"[tiab] OR "developing economies"[tiab] OR "less developed economy"[tiab] OR "less developed economies"[tiab] OR "lesser developed economy"[tiab] OR "lesser developed economies"[tiab] OR "under developed economy"[tiab] OR "under developed economies"[tiab] OR "underdeveloped economy"[tiab] OR "underdeveloped economies"[tiab] OR "middle income economy"[tiab] OR "middle income economies"[tiab] OR "low income economy"[tiab] OR "low income economies"[tiab] OR "lower income economy"[tiab] OR "lower income economies"[tiab] OR "low gdp"[tiab] OR "low gnp"[tiab] OR "low gross domestic"[tiab] OR "low gross national"[tiab] OR "lower gdp"[tiab] OR "lower gnp"[tiab] OR "lower gross domestic"[tiab] OR "lower gross national"[tiab] OR lmic[tiab] OR lmics[tiab] OR "third world"[tiab] OR "lami country"[tiab] OR "lami countries"[tiab] OR "transitional country"[tiab] OR "transitional countries"[tiab] OR Africa[tiab] OR Asia[tiab] OR Caribbean[tiab] OR “West Indies”[tiab] OR “South America”[tiab] OR “Latin America”[tiab] OR “Central America”[tiab] OR “Afghanistan”[tiab] OR Angola[tiab] OR Armenia[tiab] OR Armenian[tiab] OR Bangladesh[tiab] OR Benin[tiab] OR Byelarus[tiab] OR Byelorussian[tiab] OR Belorussian[tiab] OR Belorussia[tiab] OR Bhutan[tiab] OR Bolivia[tiab] OR “Burkina Faso”[tiab] OR “Burkina Fasso”[tiab] OR “Upper Volta”[tiab] OR Burundi[tiab] OR Urundi[tiab] OR Cambodia[tiab] OR “Khmer Republic”[tiab] OR Kampuchea[tiab] OR Cameroon[tiab] OR Cameroons[tiab] OR Cameron[tiab] OR Camerons[tiab] OR “Cape Verde”[tiab] OR “Central African Republic”[tiab] OR Chad[tiab] OR Comoros[tiab] OR Comoro Islands[tiab] OR Comores[tiab] OR Mayotte[tiab] OR Congo[tiab] OR Zaire[tiab] OR “Cote d'Ivoire”[tiab] OR “Ivory Coast”[tiab] OR Cyprus[tiab] OR Czechoslovakia[tiab] OR Djibouti[tiab] OR “French Somaliland”[tiab] OR “East Timor”[tiab] OR “East Timur”[tiab] OR “Timor Leste”[tiab] OR Egypt[tiab] OR “El Salvador”[tiab] OR Eritrea[tiab] OR Ethiopia[tiab] OR “Gabonese Republic”[tiab] OR Gambia[tiab] OR Gaza[tiab] OR “Georgia Republic”[tiab] OR “Georgian Republic”[tiab] OR Ghana[tiab] OR “Gold Coast”[tiab] OR Guatemala[tiab] OR Guinea[tiab] OR Guiana[tiab] OR Haiti[tiab] OR Honduras[tiab] OR India[tiab] OR Indonesia[tiab] OR Jordan[tiab] OR Kazakh[tiab] OR Kenya[tiab] OR Kiribati[tiab] OR Kosovo[tiab] OR Kyrgyzstan[tiab] OR Kirghizia[tiab] OR “Kyrgyz Republic”[tiab] OR Kirghiz[tiab] OR Kirgizstan[tiab] OR "Lao PDR"[tiab] OR Laos[tiab] OR Lesotho[tiab] OR Basutoland[tiab] OR Liberia[tiab] OR Madagascar[tiab] OR “Malagasy Republic”[tiab] OR Malaya[tiab] OR Malay[tiab] OR Sabah[tiab] OR Sarawak[tiab] OR Malawi[tiab] OR Nyasaland[tiab] OR Mali[tiab] OR Mauritania[tiab] OR “Agalega Islands”[tiab] OR Micronesia[tiab] OR “Middle East”[tiab] OR Moldova[tiab] OR Moldovia[tiab] OR Moldovian[tiab] OR Mongolia[tiab] OR Morocco[tiab] OR Ifni[tiab] OR Mozambique[tiab] OR Myanmar[tiab] OR Myanma[tiab] OR Burma[tiab] OR Nepal[tiab] OR Nicaragua[tiab] OR Niger[tiab] OR Nigeria[tiab] OR Muscat[tiab] OR Pakistan[tiab] OR Palestine[tiab] OR Philippines[tiab] OR Philipines[tiab] OR Phillipines[tiab] OR Phillippines[tiab] OR Rumania[tiab] OR Roumania[tiab] OR Russia[tiab] OR Russian[tiab] OR Rwanda[tiab] OR Ruanda[tiab] OR “Navigator Island”[tiab] OR “Navigator Islands”[tiab] OR “Sao Tome”[tiab] OR Senegal[tiab] OR “Sierra Leone”[tiab] OR “Sri Lanka”[tiab] OR Ceylon[tiab] OR “Solomon Islands”[tiab] OR Somalia[tiab] OR Sudan[tiab] OR Swaziland[tiab] OR Syria[tiab] OR Tajikistan[tiab] OR Tadzhikistan[tiab] OR Tadjikistan[tiab] OR Tadzhik[tiab] OR Tanzania[tiab] OR Togo[tiab] OR “Togolese Republic”[tiab] OR Tunisia[tiab] OR Turkmen[tiab] OR Uganda[tiab] OR Ukraine[tiab] OR USSR[tiab] OR “Soviet Union”[tiab] OR “Union of Soviet Socialist Republics”[tiab] OR Uzbekistan[tiab] OR Uzbek OR Vanuatu[tiab] OR “New Hebrides”[tiab] OR Vietnam[tiab] OR “Viet Nam”[tiab] OR “West Bank”[tiab] OR Yemen[tiab] OR Yugoslavia[tiab] OR Zambia[tiab] OR Zimbabwe[tiab] OR Rhodesia[tiab] OR “Developing Countries”[Mesh:noexp] OR Africa[Mesh:noexp] OR “Africa, Northern”[Mesh:noexp] OR “Africa South of the Sahara”[Mesh:noexp] OR “Africa, Central”[Mesh:noexp] OR “Africa, Eastern”[Mesh:noexp] OR “Africa, Southern”[Mesh:noexp] OR “Africa, Western”[Mesh:noexp] OR Asia[Mesh:noexp] OR “Asia, Central”[Mesh:noexp] OR “Asia, Southeastern”[Mesh:noexp] OR “Asia, Western”[Mesh:noexp] OR “Caribbean Region”[Mesh:noexp] OR “West Indies”[Mesh:noexp] OR “South America”[Mesh:noexp] OR “Latin America”[Mesh:noexp] OR “Central America”[Mesh:noexp] OR Afghanistan[Mesh:noexp] OR Angola[Mesh:noexp] OR Armenia[Mesh:noexp] OR Bangladesh[Mesh:noexp] OR Benin[Mesh:noexp] OR Byelarus[Mesh:noexp] OR Bhutan[Mesh:noexp] OR Bolivia[Mesh:noexp] OR “Burkina Faso”[Mesh:noexp] OR Burundi[Mesh:noexp] OR Cambodia[Mesh:noexp] OR Cameroon[Mesh:noexp] OR “Cape Verde”[Mesh:noexp] OR “Central African Republic”[Mesh:noexp] OR Chad[Mesh:noexp] OR Comoros[Mesh:noexp] OR Congo[Mesh:noexp] OR “Cote d'Ivoire”[Mesh:noexp] OR Cyprus[Mesh:noexp] OR Czechoslovakia[Mesh:noexp] OR Djibouti[Mesh:noexp] OR "Democratic Republic of the Congo"[Mesh:noexp] OR “East Timor”[Mesh:noexp] OR Egypt[Mesh:noexp] OR “El Salvador”[Mesh:noexp] OR Eritrea[Mesh:noexp] OR Ethiopia[Mesh:noexp] OR Gambia[Mesh:noexp] OR "Georgia (Republic)"[Mesh:noexp] OR Ghana[Mesh:noexp] OR Guatemala[Mesh:noexp] OR Guinea[Mesh:noexp] OR Guinea-Bissau[Mesh:noexp] OR Haiti[Mesh:noexp] OR Honduras[Mesh:noexp] OR India[Mesh:noexp] OR Indonesia[Mesh:noexp] OR Jordan[Mesh:noexp] OR Kenya[Mesh:noexp] OR Kosovo[Mesh:noexp] OR Kyrgyzstan[Mesh:noexp] OR Laos[Mesh:noexp] OR Lesotho[Mesh:noexp] OR Liberia[Mesh:noexp] OR Madagascar[Mesh:noexp] OR Malawi[Mesh:noexp] OR Mali[Mesh:noexp] OR Mauritania[Mesh:noexp] OR Micronesia[Mesh:noexp] OR “Middle East”[Mesh:noexp] OR Moldova[Mesh:noexp] OR Mongolia[Mesh:noexp] OR Montenegro[Mesh:noexp] OR Morocco[Mesh:noexp] OR Mozambique[Mesh:noexp] OR Myanmar[Mesh:noexp] OR Nepal[Mesh:noexp] OR Nicaragua[Mesh:noexp] OR Niger[Mesh:noexp] OR Nigeria[Mesh:noexp] OR Pakistan[Mesh:noexp] OR “Papua New Guinea”[Mesh:noexp] OR Philippines[Mesh:noexp] OR Russia[Mesh:noexp] OR "Russia (Pre-1917)"[Mesh:noexp] OR Rwanda[Mesh:noexp] OR Senegal[Mesh:noexp] OR Montenegro[Mesh:noexp] OR “Sierra Leone”[Mesh:noexp] OR “Sri Lanka”[Mesh:noexp] OR Somalia[Mesh:noexp] OR Sudan[Mesh:noexp] OR Swaziland[Mesh:noexp] OR Syria[Mesh:noexp] OR Tajikistan[Mesh:noexp] OR Tanzania[Mesh:noexp] OR Togo[Mesh:noexp] OR Tunisia[Mesh:noexp] OR Uganda[Mesh:noexp] OR Ukraine[Mesh:noexp] OR USSR[Mesh:noexp] OR Uzbekistan[Mesh:noexp] OR Vanuatu[Mesh:noexp] OR Vietnam[Mesh:noexp] OR Yemen[Mesh:noexp] OR Yugoslavia[Mesh:noexp] OR Zambia[Mesh:noexp] OR Zimbabwe[Mesh:noexp]

Concept #3: Access Barriers

"Health Services Accessibility"[Mesh] OR "Health Equity"[Mesh] OR ((“afford”[tiab] OR “affordable”[tiab] OR “affordability”[tiab] or “sustain”[tiab] OR “equal”[tiab] or “inequal”[tiab] OR “barrier”[tiab] OR “barriers”[tiab] OR “obstacle”[tiab] OR “obstacles”[tiab] OR “inequities”[tiab] OR “equitable”[tiab] OR “inequitable”[tiab] OR “equity”[tiab] OR “inequity”[tiab]) AND (“access”[tiab])) OR ((“constrain”[tiab] OR “constrained”[tiab] OR “constraint”[tiab] OR restrict*[tiab] OR “limited”[tiab] OR “reduced”[tiab] OR “reduce”[tiab]) AND (“cost”[tiab] OR “costs”[tiab] OR “consent”[tiab] OR “transportation”[tiab] OR “transport”[tiab])) OR “stigma”[tiab] OR “cultural norm”[tiab] OR “cultural norms”[tiab]

Concept #4: Health Services

“health care”[tiab] OR “healthcare”[tiab] OR “health services”[tiab] OR "Adolescent Health Services"[Mesh] OR "Reproductive Health"[Mesh] OR "Sexual Health"[Mesh] OR “reproductive health”[tiab] OR “sexual health”[tiab] OR “contraception”[tiab] OR “contraceptive”[tiab] OR “contraceptives”[tiab] OR “menstruation”[tiab] OR “mental health”[tiab] OR “HPV”[tiab] OR “human papillomavirus”[tiab] OR “human papillomaviruses”[tiab]

Concept #5: Evidence Filter

"cohort studies"[mesh:noexp] OR "cohort"[tiab] OR "cohorts"[tiab] OR "concurrent"[tiab] OR "follow up"[tiab] OR "follow-up studies"[mesh] OR "followup"[tiab] OR "incidence"[tiab] OR "long term"[tiab] OR "longitudinal studies"[mesh] OR "longitudinal"[tiab] OR "longterm"[tiab] OR "multi center"[tiab] OR "multi centre"[tiab] OR "multicenter studies as topic"[mesh] OR "multicenter"[tiab] OR "multicentre"[tiab] OR "non experimental"[tiab] OR "nonexperimental"[tiab] OR "observational studies as topic"[mesh] OR "observational study"[pt] OR "phase 1"[tiab] OR "phase 2"[tiab] OR "phase 3"[tiab] OR "phase 4"[tiab] OR "phase four"[tiab] OR "phase i"[tiab] OR "phase ii"[tiab] OR "phase iii"[tiab] OR "phase iv"[tiab] OR "phase one"[tiab] OR "phase three"[tiab] OR "phase two"[tiab] OR "phase four"[tiab] OR "prospective studies"[mesh] OR "randomisation"[tiab] OR "randomization"[tiab] OR "randomized" [tiab] OR "randomised"[tiab] OR "randomly" [tiab] OR "random"[tiab] OR "studies"[ti] OR "study"[ti] OR drug therapy[sh] OR groups [tiab] OR observation*[tiab] OR placebo [tiab] OR prospective*[tiab] OR randomized[tiab] OR randomly[tiab] OR systematic*[tiab] OR trial* [ti] OR "meta analysis"[tiab] OR "metaanalysis"[tiab] OR "meta analyses"[tiab] OR "metaanalyses"[tiab] OR “focus group”[tiab] OR “focus groups”[tiab] OR interview*[tiab] OR ethnograph*[tiab] OR narrative*[tiab] OR “participatory action”[tiab] OR qualitative[tiab] OR qualitative[tiab] OR survey*[tiab] OR model*[tiab] OR multi-level*[tiab] OR “Delphi design”[tiab] OR case-control[tiab] OR review[tiab] OR phenomenol*[tiab] OR “network analysis”[tiab] OR “mixed methods”[tiab] OR “mixed-methods”[tiab] OR “facility assessment”[tiab]

Exclusion Filter:

NOT

“under 5”[tiab] OR “under-5”[tiab] OR “maternal and child”[tiab] OR “under the age of five”[tiab] OR “under the age of 5”[tiab] OR “younger than 5”[tiab] OR “younger than age 5”[tiab] OR "Child Care"[Mesh] OR "Child Day Care Centers"[Mesh] OR neonat*[tiab] OR baby[tiab] OR babies[tiab] OR pediatric[tiab] OR paediatric[tiab] OR "Maternal Health Services"[Mesh] OR "Maternal Health"[Mesh]

Inversion filter:

NOT "Child"[Mesh] NOT ((“Adolescent”[Mesh] OR pre-adolescent[tiab] OR adolescent*[tiab]) AND (“Child”[Mesh]))

______________________

**Embase: Initial Search 9/26/18; Update 10/14/2019 Dates Limited 2005-2019**

Concept #1: Adolescent

‘adolescent’:ab,ti OR ‘adolescents’:ab,ti OR ‘adolescence’:ab,ti OR ‘teenage’:ab,ti OR ‘teenager’:ab,ti OR ‘teenagers’:ab,ti OR ‘youth’:ab,ti OR ‘youths’:ab,ti OR ‘young people’:ab,ti OR ‘girls’:ab,ti

Concept #2: Developing Countries

‘developing country’:ab,ti OR ‘developing countries’:ab,ti OR ‘developing nation’:ab,ti OR ‘developing nations’:ab,ti OR ‘developing population’:ab,ti OR ‘developing populations’:ab,ti OR ‘developing world’:ab,ti OR ‘less developed country’:ab,ti OR ‘less developed countries’:ab,ti OR ‘less developed nation’:ab,ti OR ‘less developed nations’:ab,ti OR ‘less developed population’:ab,ti OR ‘less developed populations’:ab,ti OR ‘less developed world’:ab,ti OR ‘lesser developed country’:ab,ti OR ‘lesser developed countries’:ab,ti OR ‘lesser developed nation’:ab,ti OR ‘lesser developed nations’:ab,ti OR ‘lesser developed population’:ab,ti OR ‘lesser developed populations’:ab,ti OR ‘lesser developed world’:ab,ti OR ‘under developed country’:ab,ti OR ‘under developed countries’:ab,ti OR ‘under developed nation’:ab,ti OR ‘under developed nations’:ab,ti OR ‘under developed population’:ab,ti OR ‘under developed populations’:ab,ti OR ‘under developed world’:ab,ti OR ‘underdeveloped country’:ab,ti OR ‘underdeveloped countries’:ab,ti OR ‘underdeveloped nation’:ab,ti OR ‘underdeveloped nations’:ab,ti OR ‘underdeveloped population’:ab,ti OR ‘underdeveloped populations’:ab,ti OR ‘underdeveloped world’:ab,ti OR ‘middle income country’:ab,ti OR ‘middle income countries’:ab,ti OR ‘middle income nation’:ab,ti OR ‘middle income nations’:ab,ti OR ‘middle income population’:ab,ti OR ‘middle income populations’:ab,ti OR ‘middle-income settings’:ab,ti OR ‘low-income settings’:ab,ti OR ‘low income country’:ab,ti OR ‘low income countries’:ab,ti OR ‘low income nation’:ab,ti OR ‘low income nations’:ab,ti OR ‘low income population’:ab,ti OR ‘low income populations’:ab,ti OR ‘lower income country’:ab,ti OR ‘lower income countries’:ab,ti OR ‘lower income nation’:ab,ti OR ‘lower income nations’:ab,ti OR ‘lower income population’:ab,ti OR ‘lower income populations’:ab,ti OR ‘underserved country’:ab,ti OR ‘underserved countries’:ab,ti OR ‘underserved nation’:ab,ti OR ‘underserved nations’:ab,ti OR ‘underserved population’:ab,ti OR ‘underserved populations’:ab,ti OR ‘underserved world’:ab,ti OR ‘under served country’:ab,ti OR ‘under served countries’:ab,ti OR ‘under served nation’:ab,ti OR ‘under served nations’:ab,ti OR ‘under served population’:ab,ti OR ‘under served populations’:ab,ti OR ‘under served world’:ab,ti OR ‘deprived country’:ab,ti OR ‘deprived countries’:ab,ti OR ‘deprived nation’:ab,ti OR ‘deprived nations’:ab,ti OR ‘deprived population’:ab,ti OR ‘deprived populations’:ab,ti OR ‘deprived world’:ab,ti OR ‘poor country’:ab,ti OR ‘poor countries’:ab,ti OR ‘poor nation’:ab,ti OR ‘poor nations’:ab,ti OR ‘poor population’:ab,ti OR ‘poor populations’:ab,ti OR ‘poor world’:ab,ti OR ‘poorer country’:ab,ti OR ‘poorer countries’:ab,ti OR ‘poorer nation’:ab,ti OR ‘poorer nations’:ab,ti OR ‘poorer population’:ab,ti OR ‘poorer populations’:ab,ti OR ‘poorer world’:ab,ti OR ‘developing economy’:ab,ti OR ‘developing economies’:ab,ti OR ‘less developed economy’:ab,ti OR ‘less developed economies’:ab,ti OR ‘lesser developed economy’:ab,ti OR ‘lesser developed economies’:ab,ti OR ‘under developed economy’:ab,ti OR ‘under developed economies’:ab,ti OR ‘underdeveloped economy’:ab,ti OR ‘underdeveloped economies’:ab,ti OR ‘middle income economy’:ab,ti OR ‘middle income economies’:ab,ti OR ‘low income economy’:ab,ti OR ‘low income economies’:ab,ti OR ‘lower income economy’:ab,ti OR ‘lower income economies’:ab,ti OR ‘low gdp’:ab,ti OR ‘low gnp’:ab,ti OR ‘low gross domestic’:ab,ti OR ‘low gross national’:ab,ti OR ‘lower gdp’:ab,ti OR ‘lower gnp’:ab,ti OR ‘lower gross domestic’:ab,ti OR ‘lower gross national’:ab,ti OR ‘lmic’:ab,ti OR ‘lmics’:ab,ti OR ‘third world’:ab,ti OR ‘lami country’:ab,ti OR ‘lami countries’:ab,ti OR ‘transitional country’:ab,ti OR ‘transitional countries’:ab,ti OR ‘Africa’:ab,ti OR ‘Asia’:ab,ti OR ‘Caribbean’:ab,ti OR ‘West Indies’:ab,ti OR ‘South America’:ab,ti OR ‘Latin America’:ab,ti OR ‘Central America’:ab,ti OR ‘Afghanistan’:ab,ti OR ‘Angola’:ab,ti OR ‘Armenia’:ab,ti OR ‘Armenian’:ab,ti OR ‘Bangladesh’:ab,ti OR ‘Benin’:ab,ti OR ‘Byelarus’:ab,ti OR ‘Byelorussian’:ab,ti OR ‘Belorussian’:ab,ti OR ‘Belorussia’:ab,ti OR ‘Bhutan’:ab,ti OR ‘Bolivia’:ab,ti OR ‘Burkina Faso’:ab,ti OR ‘Burkina Fasso’:ab,ti OR ‘Upper Volta’:ab,ti OR ‘Burundi’:ab,ti OR ‘Urundi’:ab,ti OR ‘Cambodia’:ab,ti OR ‘Khmer Republic’:ab,ti OR ‘Kampuchea’:ab,ti OR ‘Cameroon’:ab,ti OR ‘Cameroons’:ab,ti OR ‘Cameron’:ab,ti OR ‘Camerons’:ab,ti OR ‘Cape Verde’:ab,ti OR ‘Central African Republic’:ab,ti OR ‘Chad’:ab,ti OR ‘Comoros’:ab,ti OR ‘Comoro Islands’:ab,ti OR ‘Comores’:ab,ti OR ‘Mayotte’:ab,ti OR ‘Congo’:ab,ti OR ‘Zaire’:ab,ti OR ‘Ivory Coast’:ab,ti OR ‘Cyprus’:ab,ti OR ‘Czechoslovakia’:ab,ti OR ‘Djibouti’:ab,ti OR ‘French Somaliland’:ab,ti OR ‘East Timor’:ab,ti OR ‘East Timur’:ab,ti OR ‘Timor Leste’:ab,ti OR ‘Egypt’:ab,ti OR ‘El Salvador’:ab,ti OR ‘Eritrea’:ab,ti OR ‘Ethiopia’:ab,ti OR ‘Gabonese Republic’:ab,ti OR ‘Gambia’:ab,ti OR ‘Gaza’:ab,ti OR ‘Georgia Republic’:ab,ti OR ‘Georgian Republic’:ab,ti OR ‘Ghana’:ab,ti OR ‘Gold Coast’:ab,ti OR ‘Guatemala’:ab,ti OR ‘Guinea’:ab,ti OR ‘Guiana’:ab,ti OR ‘Haiti’:ab,ti OR ‘Honduras’:ab,ti OR ‘India’:ab,ti OR ‘Indonesia’:ab,ti OR ‘Jordan’:ab,ti OR ‘Kazakh’:ab,ti OR ‘Kenya’:ab,ti OR ‘Kiribati’:ab,ti OR ‘Kosovo’:ab,ti OR ‘Kyrgyzstan’:ab,ti OR ‘Kirghizia’:ab,ti OR ‘Kyrgyz Republic’:ab,ti OR ‘Kirghiz’:ab,ti OR ‘Kirgizstan’:ab,ti OR ‘Lao PDR’:ab,ti OR ‘Laos’:ab,ti OR ‘Lesotho’:ab,ti OR ‘Basutoland’:ab,ti OR ‘Liberia’:ab,ti OR ‘Madagascar’:ab,ti OR ‘Malagasy Republic’:ab,ti OR ‘Malaya’:ab,ti OR ‘Malay’:ab,ti OR ‘Sabah’:ab,ti OR ‘Sarawak’:ab,ti OR ‘Malawi’:ab,ti OR ‘Nyasaland’:ab,ti OR ‘Mali’:ab,ti OR ‘Mauritania’:ab,ti OR ‘Agalega Islands’:ab,ti OR ‘Micronesia’:ab,ti OR ‘Middle East’:ab,ti OR ‘Moldova’:ab,ti OR ‘Moldovia’:ab,ti OR ‘Moldovian’:ab,ti OR ‘Mongolia’:ab,ti OR ‘Morocco’:ab,ti OR ‘Ifni’:ab,ti OR ‘Mozambique’:ab,ti OR ‘Myanmar’:ab,ti OR ‘Myanma’:ab,ti OR ‘Burma’:ab,ti OR ‘Nepal’:ab,ti OR ‘Nicaragua’:ab,ti OR ‘Niger’:ab,ti OR ‘Nigeria’:ab,ti OR ‘Muscat’:ab,ti OR ‘Pakistan’:ab,ti OR ‘Palestine’:ab,ti OR ‘Philippines’:ab,ti OR ‘Philipines’:ab,ti OR ‘Phillipines’:ab,ti OR ‘Phillippines’:ab,ti OR ‘Rumania’:ab,ti OR ‘Roumania’:ab,ti OR ‘Russia’:ab,ti OR ‘Russian’:ab,ti OR ‘Rwanda’:ab,ti OR ‘Ruanda’:ab,ti OR ‘Navigator Island’:ab,ti OR ‘Navigator Islands’:ab,ti OR ‘Sao Tome’:ab,ti OR ‘Senegal’:ab,ti OR ‘Sierra Leone’:ab,ti OR ‘Sri Lanka’:ab,ti OR ‘Ceylon’:ab,ti OR ‘Solomon Islands’:ab,ti OR ‘Somalia’:ab,ti OR ‘Sudan’:ab,ti OR ‘Swaziland’:ab,ti OR ‘Syria’:ab,ti OR ‘Tajikistan’:ab,ti OR ‘Tadzhikistan’:ab,ti OR ‘Tadjikistan’:ab,ti OR ‘Tadzhik’:ab,ti OR ‘Tanzania’:ab,ti OR ‘Togo’:ab,ti OR ‘Togolese Republic’:ab,ti OR ‘Tunisia’:ab,ti OR ‘Turkmen’:ab,ti OR ‘Uganda’:ab,ti OR ‘Ukraine’:ab,ti OR ‘USSR’:ab,ti OR ‘Soviet Union’:ab,ti OR ‘Union of Soviet’ OR ‘Socialist Republics’:ab,ti OR ‘Uzbekistan’:ab,ti OR ‘Uzbek’:ab,ti OR ‘Vanuatu’:ab,ti OR ‘New Hebrides’:ab,ti OR ‘Vietnam’:ab,ti OR ‘Viet Nam’:ab,ti OR ‘West Bank’:ab,ti OR ‘Yemen’:ab,ti OR ‘Yugoslavia’:ab,ti OR ‘Zambia’:ab,ti OR ‘Zimbabwe’:ab,ti OR ‘Rhodesia’:ab,ti OR ‘Developing Country’/de OR ‘Africa’/de 'North Africa'/de OR ‘Africa South of the Sahara’/de OR 'Central Africa'/de OR ‘Southern Africa’/de OR ‘West Africa’/de OR ‘Asia’/de OR ‘South Asia’/de OR ‘Caribbean’/de OR ‘Caribbean Islands’/de OR ‘South America’/de OR 'South and Central America'/de OR ‘Central America’/de OR ‘Afghanistan’/de OR ‘Angola’/de OR ‘Armenia’/de OR ‘Bangladesh’/de OR ‘Benin’/de OR ‘Belarus’/de OR ‘Bhutan’/de OR ‘Bolivia’/de OR ‘Burkina Faso’/de OR ‘Burundi’/de OR ‘Cambodia’/de OR ‘Cameroon’/de OR ‘Cape Verde’/de OR ‘Central African Republic’/de OR ‘Chad’/de OR ‘Comoros’/de OR ‘Congo’/de OR ‘Cyprus’/de OR ‘Czechoslovakia’/de OR ‘Djibouti’/de OR ‘Democratic Republic of the Congo’/de OR 'Timor-Leste'/de OR ‘Egypt’/de OR ‘El Salvador’/de OR ‘Eritrea’/de OR ‘Ethiopia’/de OR ‘Gambia’/de OR ‘Georgia (Republic)’/de OR ‘Ghana’/de OR ‘Guatemala’/de OR ‘Guinea’/de OR ‘Guinea-Bissau’/de OR ‘Haiti’/de OR ‘Honduras’/de OR ‘India’/de OR ‘Indonesia’/de OR ‘Jordan’/de OR ‘Kenya’/de OR ‘Kosovo’/de OR ‘Kyrgyzstan’/de OR ‘Laos’/de OR ‘Lesotho’/de OR ‘Liberia’/de OR ‘Madagascar’/de OR ‘Malawi’/de OR ‘Mali’/de OR ‘Mauritania’/de OR 'Federated States of Micronesia'/de OR ‘Middle East’/de OR ‘Moldova’/de OR ‘Mongolia’/de OR ‘Montenegro (republic)’/de OR ‘Morocco’/de OR ‘Mozambique’/de OR ‘Myanmar’/de OR ‘Nepal’/de OR ‘Nicaragua’/de OR ‘Niger’/de OR ‘Nigeria’/de OR ‘Pakistan’/de OR ‘Papua New Guinea’/de OR ‘Philippines’/de OR ‘Russian Federation’/de OR ‘USSR’/de OR ‘Rwanda’/de OR ‘Senegal’/de OR ‘Sierra Leone’/de OR ‘Sri Lanka’/de OR ‘Somalia’/de OR ‘Sudan’/de OR ‘Swaziland’/de OR ‘Syria’/de OR ‘Tajikistan’/de OR ‘Tanzania’/de OR ‘Togo’/de OR ‘Tunisia’/de OR ‘Uganda’/de OR ‘Ukraine’/de OR ‘USSR’/de OR ‘Uzbekistan’/de OR ‘Vanuatu’/de OR ‘Viet nam’/de OR ‘Yemen’/de OR ‘Yugoslavia’/de OR ‘Zambia’/de OR ‘Zimbabwe’/de

Concept #3: Access Barriers

'access to care'/exp OR 'health equity'/exp OR ((‘afford’:ab,ti OR ‘affordable’:ab,ti OR ‘affordability’:ab,ti or ‘equal’:ab,ti or ‘inequal’:ab,ti OR ‘barrier’:ab,ti OR ‘barriers’:ab,ti OR ‘obstacle’:ab,ti OR ‘obstacles’:ab,ti OR ‘inequities’:ab,ti OR ‘equitable’:ab,ti OR ‘inequitable’:ab,ti OR ‘equity’:ab,ti OR ‘inequity’:ab,ti) AND (‘access’:ab,ti)) OR ((‘constrain’:ab,ti OR ‘constrained’:ab,ti OR ‘constraint’:ab,ti OR restrict*:ab,ti OR ‘limited’:ab,ti OR ‘reduced’:ab,ti OR ‘reduce’:ab,ti) AND (‘cost’:ab,ti OR ‘costs’:ab,ti OR ‘consent’:ab,ti OR ‘transportation’:ab,ti OR ‘transport’:ab,ti)) OR ‘stigma’:ab,ti OR ‘cultural norm’:ab,ti OR ‘cultural norms’:ab,ti

Concept #4: Health Services

'adolescent health'/exp OR 'reproductive health'/exp OR 'sexual health'/exp OR ‘health care’:ab,ti OR ‘healthcare’:ab,ti OR ‘health services’:ab,ti OR ‘reproductive health’:ab,ti OR ‘sexual health’:ab,ti OR ‘contraception’:ab,ti OR ‘contraceptive’:ab,ti OR ‘contraceptives’:ab,ti OR ‘menstruation’:ab,ti OR ‘mental health’:ab,ti OR ‘HPV’:ab,ti OR ‘human papillomavirus’:ab,ti OR ‘human papillomaviruses’:ab,ti

Concept #5: Evidence Filter

‘cohort’:ab,ti OR ‘cohorts’:ab,ti OR ‘concurrent’:ab,ti OR ‘follow up’:ab,ti OR ‘followup’:ab,ti OR ‘incidence’:ab,ti OR ‘long term’:ab,ti OR ‘longitudinal’:ab,ti OR ‘longterm’:ab,ti OR ‘multi center’:ab,ti OR ‘multi centre’:ab,ti OR ‘multicenter’:ab,ti OR ‘multicentre’:ab,ti OR ‘non experimental’:ab,ti OR ‘nonexperimental’:ab,ti OR ‘phase 1’:ab,ti OR ‘phase 2’:ab,ti OR ‘phase 3’:ab,ti OR ‘phase 4’:ab,ti OR ‘phase four’:ab,ti OR ‘phase i’:ab,ti OR ‘phase ii’:ab,ti OR ‘phase iii’:ab,ti OR ‘phase iv’:ab,ti OR ‘phase one’:ab,ti OR ‘phase three’:ab,ti OR ‘phase two’:ab,ti OR ‘phase four’:ab,ti OR ‘randomisation’:ab,ti OR ‘randomization’:ab,ti OR ‘randomized’:ab,ti OR ‘randomised’:ab,ti OR ‘randomly’:ab,ti OR ‘random’:ab,ti OR ‘studies’:ti OR ‘study’:ti OR ‘groups’:ab,ti OR ‘observation*’:ab,ti OR ‘placebo’:ab,ti OR ‘prospective*’:ab,ti OR ‘randomized’:ab,ti OR ‘randomly’:ab,ti OR ‘systematic*’:ab,ti OR ‘trial*’:ti OR ‘meta analysis’:ab,ti OR ‘metaanalysis’:ab,ti OR ‘meta analyses’:ab,ti OR ‘metaanalyses’:ab,ti OR ‘focus group’:ab,ti OR ‘focus groups’:ab,ti OR ‘interview*’:ab,ti OR ‘ethnograph*’:ab,ti OR ‘narrative*’:ab,ti OR ‘participatory action’:ab,ti OR ‘qualitative’:ab,ti OR ‘qualitative’:ab,ti OR ‘survey*’:ab,ti OR ‘model*’:ab,ti OR ‘multi-level*’:ab,ti OR ‘Delphi design’:ab,ti OR ‘case-control’:ab,ti OR ‘review’:ab,ti OR ‘phenomenol*’:ab,ti OR ‘network analysis’:ab,ti OR ‘mixed methods’:ab,ti OR ‘mixed-methods’:ab,ti OR ‘facility assessment’:ab,ti OR 'cohort analysis'/exp OR 'follow up'/exp OR 'longitudinal study'/exp OR 'observational study'/exp OR 'multicenter study'/exp OR 'drug therapy'/mj

Exclusion Filter:

NOT ‘under 5’:ab,ti OR ‘under-5’:ab,ti OR ‘maternal and child’:ab,ti OR 'under the age of five’:ab,ti OR ‘under the age of 5’:ab,ti OR ‘younger than 5’:ab,ti OR ‘younger than age 5’:ab,ti OR 'child care'/exp OR 'day care'/exp OR ‘neonat*’:ab,ti OR ‘baby’:ab,ti OR ‘babies’:ab,ti OR ‘pediatric’:ab,ti OR ‘paediatric’:ab,ti OR 'maternal health service'/exp OR ‘Maternal Health’:ab,ti

Inversion filter:

NOT 'child'/exp NOT (('adolescent'/exp OR ‘pre-adolescent’:ab,ti OR ‘adolescent*’:ab,ti) AND (‘child’/exp))
