# Supplementary material for: Assessing Barriers to Effective Coverage of Health Services for Adolescents in Low- and Middle-Income Countries: A Scoping Review
Source: J Adolesc Health. 2021 Oct;69(4):541–8. doi: 10.1016/j.jadohealth.2020.12.135 (PMC8442758; doi:10.1016/j.jadohealth.2020.12.135)
Supplement: Table S1 [file mmc3.docx]

Table S1. Operational Framework for Barriers on the Pathway from Access to Effective Health Coverage

| **Health coverage dimensions** | **Supply side** | **Demand side** |
| --- | --- | --- |
| **Availability** | - Skilled health workers, staff absenteeism, - Opening hours, appointments systems - Waiting time - Motivation of staff - Drugs and other consumables; equipment - Service mix provided - Non-integration of health services - Lack of opportunity (exclusion from services) - Late or no referral - Poor technical quality of intervention - Provider compliance with intervention - Diagnostic accuracy | - Information on health care services/providers - Education/knowledge about services and health needs |
| **Accessibility** (Geographic) | - Service location; distance, time to services | - Means of transport available - Transport costs to household |
| **Affordability** | - Costs and prices of services, including informal payments - Private–public dual practice - Fiscal space and public sector financial commitment to health | - Household resources and willingness to pay - Opportunity costs (e.g. lost work time, child care) - Cash availability (and prior impoverishing expenditure) |
| **Acceptability** | - Staff interpersonal skills - Power relationships to patients - Age/sex appropriateness - Discrimination (based on gender, ethnicity, religion, caste, race, sexual orientation, age, language, ability, disease) - Complexity of billing system and inability for patients to know prices beforehand | - Household expectations, trust - Low self-esteem and little assertiveness - Community and cultural preferences (including gender norms) - Stigma - Lack of health awareness - Confidentiality expected - Perceptions of service quality - Adherence to treatment barriers (including those related to interaction with provider; power/gender imbalance) |
| **Utilization** (Contact coverage; a product of Availability 🡪 Acceptability) | | |
| **Effective Coverage**  The estimate of the fraction (or rate) of **potential health gain** that is actually **delivered to the population** through the health system. | Article must use the term “effective coverage”. This is a measure of the population that **needs** the intervention (e.g. prevalence) AND the proportion (or rate) of those that need the intervention that use the service (coverage among those in need), AND the **quality** of the intervention (in this case the actual health benefit). The measure must include a measure of the population that needs the intervention (e.g. from population estimate, cohort of people with a condition or through use of biomarkers), a population-based measure of utilization (coverage), and a health outcome related to the service (not just a measure of number of services provided). | |
|  | Can be measured directly by:   - Combining population prevalence, utilization, and direct measures of patient care (i.e. diagnostic accuracy of provider and patient adherence) and health outcomes (or estimated from known efficacy if diagnostic accuracy and patient adherence are measured) - Using a (complete) registration database of people with a condition and assessing health outcomes among those receiving the intervention - Matching exposure levels of an intervention in population survey that measures outcomes - Using statistical methods or risk adjustment in household surveys (or hospital databases if all cases are treated in hospital) to estimate effect of intervention on population in need if levels of utilization are included. | |
| **Health Outcomes** (Health Status – mortality, morbidity, disability, wellbeing, or malnutrition; a product of context, genetics, opportunities & exposures, and health services) | | |

Adapted from conceptual frameworks on effective coverage and health access. (7-12)
