# Supplementary material for: Assessing Barriers to Effective Coverage of Health Services for Adolescents in Low- and Middle-Income Countries: A Scoping Review
Source: J Adolesc Health. 2021 Oct;69(4):541–8. doi: 10.1016/j.jadohealth.2020.12.135 (PMC8442758; doi:10.1016/j.jadohealth.2020.12.135)
Supplement: Table S2 [file mmc4.docx]

Table S2. Frequencies that disadvantages (or inequity stratifiers) were mentioned by health coverage dimension studied

|  | **Availability** | **Accessibility (geographic)** | **Affordability** | **Acceptability** | **Utilization** | **Effective Coverage** |
| --- | --- | --- | --- | --- | --- | --- |
|  | **n=298** | **n=108** | **n=153** | **n=314** | **n=137** | **n=0** |
| Sex | 69% | 76% | 72% | 71% | 74% | 0% |
| Rural/urban | 32% | 37% | 31% | 31% | 39% | 0% |
| Socioeconomic status | 29% | 33% | 35% | 30% | 34% | 0% |
| Marital status | 18% | 25% | 19% | 18% | 28% | 0% |
| Income | 18% | 22% | 24% | 18% | 23% | 0% |
| Education | 16% | 21% | 14% | 15% | 27% | 0% |
| Geography | 15% | 23% | 17% | 15% | 21% | 0% |
| Age | 13% | 13% | 14% | 13% | 15% | 0% |
| Out-of-school youth | 11% | 11% | 10% | 11% | 14% | 0% |
| Orphans/living arrangements | 8% | 7% | 8% | 10% | 8% | 0% |
| Occupation/employment status | 10% | 12% | 11% | 10% | 15% | 0% |
| Religion | 6% | 6% | 7% | 7% | 9% | 0% |
| Parity/childbearing status | 5% | 5% | 5% | 5% | 9% | 0% |
| Ethnicity/race | 5% | 8% | 5% | 5% | 7% | 0% |
| Youth in displaced populations | 4% | 6% | 5% | 4% | 3% | 0% |
| Developmental disability | 3% | 3% | 2% | 4% | 4% | 0% |
| Physical disability | 3% | 5% | 3% | 3% | 4% | 0% |
| Youth living in conflict areas | 3% | 5% | 4% | 3% | 2% | 0% |
| Homeless/informal housing | 3% | 3% | 3% | 3% | 3% | 0% |
| Sex industry | 2% | 3% | 2% | 2% | 4% | 0% |
| Substance abuse | 2% | 4% | 3% | 2% | 4% | 0% |
| Sexual orientation | 2% | 3% | 3% | 2% | 4% | 0% |
| Youth living in remote areas | 2% | 5% | 2% | 2% | 3% | 0% |
| Gender identity | 1% | 1% | 1% | 2% | 0% | 0% |

Note: Percentages are column percentages.

Color code: light green: >50%; light yellow: 30-49%; light orange: 10-29%; orange: 2-9%; dark orange: 0-1%.
